# Focused learning by antibody language models using preferential masking of non-templated regions

**DOI:** 10.1016/j.patter.2025.101239

**Published:** 2025-04-25

**Authors:** Karenna Ng, Bryan Briney

**Affiliations:** 1 Department of Immunology and Microbiology, The Scripps Research Institute, La Jolla, CA 92037, USA; 2 Center for Viral Systems Biology, The Scripps Research Institute, La Jolla, CA 92037, USA; 3 Multi-Omics Vaccine Evaluation Consortium, The Scripps Research Institute, La Jolla, CA 92037, USA; 4 Scripps Consortium for HIV/AIDS Vaccine Development, The Scripps Research Institute, La Jolla, CA 92037, USA; 5 San Diego Center for AIDS Research, The Scripps Research Institute, La Jolla, CA 92037, USA

**Keywords:** immunology, antibody language model, deep learning, masked language modeling, transformers

## Abstract

Existing antibody language models (AbLMs) are pre-trained using a masked language modeling (MLM) objective with uniform masking probabilities. While these models excel at predicting germline residues, they often struggle with mutated and non-templated residues, which concentrate in the complementarity-determining regions (CDRs) and are crucial for antigen binding specificity. Here, we demonstrate that preferential masking of the primarily non-templated CDR3 is a compute-efficient strategy to enhance model performance. We pre-trained two AbLMs using either uniform or preferential masking and observed that the latter improves residue prediction accuracy in the highly variable CDR3. Preferential masking also improves antibody classification by native chain pairing and binding specificity, suggesting improved CDR3 understanding and indicating that non-random, learnable patterns help govern antibody chain pairing. We further show that specificity classification is largely informed by residues in the CDRs, demonstrating that AbLMs learn meaningful patterns that align with immunological understanding.

## Highlights

• Preferential masking efficiently enhances antibody language models (AbLMs)
• Boosted CDR3 training signal improves learning of non-templated sequence patterns
• Pre-training with preferential masking improves downstream sequence classification
• Explainable AI reveals that AbLMs can learn meaningful immunological patterns

## The bigger picture

Antibodies can bind to a wide range of non-self antigens with high specificity, which is essential for immunity and has applications in assay and drug development. This ability arises from their remarkable diversity, representing a small but crucial part of the antibody sequence. Large language models hold promise for understanding the complex patterns underlying antibody structure and function. However, traditional language modeling techniques are borrowed from natural language processing and often fall short due to fundamental differences between natural language text and biological sequence data. Integrating immunological knowledge into model training could enhance performance, accelerate therapeutic development, and deepen our understanding of immunology.

## Teaser

Conventional masked language modeling (MLM) utilizes a uniform masking probability, which may not be optimal for antibody language modeling due to the unique sequence diversity patterns of antibodies. The authors developed preferential masking of the non-templated complementarity-determining region 3 (CDR3) and demonstrate that pre-training with this immunologically informed method efficiently enhances model learning and downstream sequence classification abilities.

## Introduction

Antibodies are a crucial component of the humoral immune system. Their massive diversity, estimated to be as high as 10^18^ unique antibodies,^1^ confers the potential to bind and neutralize any non-self antigen with remarkable specificity. The pre-immune antibody repertoire has combinatorial diversity from independent V(D)J recombination of both the heavy and light chains in each B cell, followed by pairing of those chains as a dimer of heterodimers.^2^^,^^3^ Each chain has three complementarity-determining regions (CDRs), which form loops that largely determine antigen binding specificity.^4^ Upon infection, antigen-specific antibodies undergo further affinity maturation, where mutations are stochastically introduced and selected for their ability to strengthen binding affinity.^5^ Although somatic mutations can be found throughout the antibody gene, they are concentrated in the CDRs.^5^^,^^6^

The amino acid sequence of a protein encodes its structure and function, analogous to how the order and context of the words in a sentence encode its meaning. This parallel has inspired the adaptation of transformer-based language models (LMs),^7^ originally developed for natural language processing (NLP), for analysis of protein sequences. Protein LMs (PLMs) have shown success at understanding evolutionary fitness^8^ as well as protein structure and function.^9^^,^^10^ Antibody LMs (AbLMs), pre-trained primarily or exclusively on antibody sequence data, have outperformed general PLMs at learning antibody-specific features such as affinity maturation,^11^^,^^12^^,^^13^ antigen specificity,^12^^,^^14^ and paratope position.^15^ However, both PLMs and AbLMs struggle to learn patterns beyond what is germline encoded. This is most evident in the poor performance in prediction of the CDR3,^12^^,^^16^^,^^17^ which spans the junction between germline V(D)J gene segments and is enriched with non-templated mutations.

Many PLMs and AbLMs use a BERT-based architecture^18^ to generate sequence embeddings that can be applied to downstream tasks such as structure prediction^10^^,^^19^ and directed evolution to improve binding affinity.^8^ These models are trained using a masked language modeling (MLM) objective, where a set of “masked” tokens are predicted based on the remaining bidirectional context.^18^ Prediction of these masked tokens, which we call “training signal,” is used to iteratively optimize the model weights. BERT randomly masks 15% of the tokens in each input sequence, and this uniform masking rate has been adopted by most BERT successors, regardless of model size or specific architecture.^9^^,^^10^^,^^11^^,^^12^^,^^13^^,^^14^^,^^15^^,^^16^^,^^20^ However, this may not be optimal for training AbLMs.

First, the 15% masking rate is thought to balance sequence corruption with training efficiency, overmasking of tokens results in limited context and poor representation learning, but pre-training is inefficient when too few tokens are predicted. However, higher masking rates have been shown to improve LM performance on multiple NLP benchmarks, especially for large-sized BERT models.^21^ Increased masking results in the model predicting more tokens for each training example, effectively learning from more of the sequence. This efficient use of training data is especially relevant for AbLMs, which are limited by a lack of paired antibody sequence training data at scale.^12^^,^^14^^,^^16^ Second, both pre-immune and affinity-matured antibody sequences consist predominantly of germline-encoded (templated) residues. Thus, under uniform masking, the majority of masked residues are in templated regions.^16^ This “frequency bias problem” limits pre-training efficiency, lowers the quality of the representations of these rare and valuable tokens,^22^^,^^23^ and makes it difficult for the model to learn non-templated patterns.

Alternative masking strategies have been explored for NLP, including changing overall masking rates^21^ and frequency-weighted token sampling.^22^ To our knowledge, few^17^ studies have applied alternative masking to AbLM training, motivating further investigation of the impacts of non-conventional pre-training on model performance. The modular nature of antibody recombination creates relatively distinct regions of high complexity (non-templated) and low complexity (templated or germline encoded). The goal of this study was to develop an alternative masking strategy based on this observation and determine to what extent AbLMs can improve when trained using this immunologically informed method.

Here, we present pre-training with preferential masking of the primarily non-templated CDR3. We hypothesized this strategy to be more informative for immunological downstream tasks, since the training signal is increased from the antibody region with the most diversity (difficult to learn) and most relevance to antigen binding. To explore this, we pre-trained two AbLMs with either uniform or preferential masking. We demonstrate that the preferential model shows enhanced ability to distinguish native chain pairings from random pairings and identify binding specificity, both of which are immunologically relevant antibody features. Using explainable artificial intelligence (XAI), we further show that classification by binding specificity is largely informed by residues in the CDRs, suggesting that our models are learning meaningful patterns that align with immunological understanding of antibody functional regions.

## Results

### Preferential masking implementation

**Figure 1:**
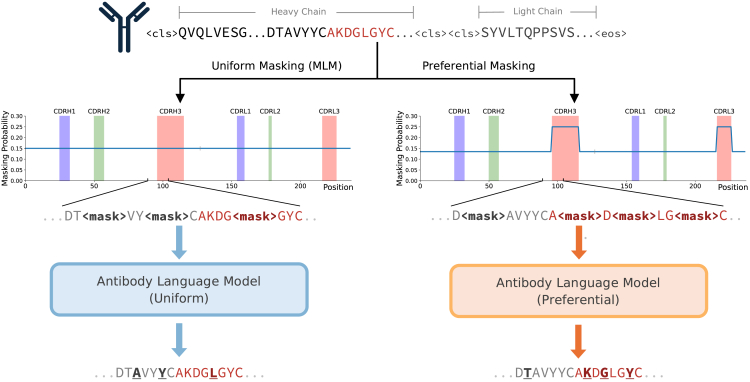
Uniform versus preferential MLM methodology Conventional masking strategies (left) employ a uniform masking probability of 15% over the entire input sequence. Preferential masking (right) increases the masking probability in the CDR3s (red) to 25% while maintaining the 15% average masking probability over the entire input sequence length.

Most models trained with an MLM objective follow the original BERT implementation, where 15% of tokens are independently and randomly selected for masking under a uniform probability distribution.^18^ Since model weights are only optimized based on masked token predictions, model performance is likely sensitive to the specific tokens that were masked. Our preferential masking strategy increases the fraction of masked tokens that come from CDR3, a diverse and primarily non-templated region that has been shown to be difficult for models to learn.^12^^,^^16^ This is achieved by increasing the masking probability to 25% in the CDR3s of both the heavy and light chains. To enable comparison against the conventional uniform masking strategy, the average masking probability across the entire input sequence length is maintained to be 15%. As a result, tokens outside of the CDR3s are masked at a rate of <15% (11.6%–14.6%). An overview of our preferential masking strategy is shown in Figure 1, and implementation details can be found in the Methods.

### Preferential masking enables more efficient model pre-training

We pre-trained two masked AbLMs using dataset A: one using conventional uniform masking and one using our preferential masking strategy. Pre-training on paired sequences was chosen to enhance learning of cross-chain features.^12^ Both used the same ESM-2-based model architecture,^10^ which includes rotary position embeddings and pre-layer normalization.^24^^,^^25^ An encoder-only architecture was chosen to allow for the generation of sequence embeddings that can be used for downstream tasks such as sequence classification.

**Figure 2:**
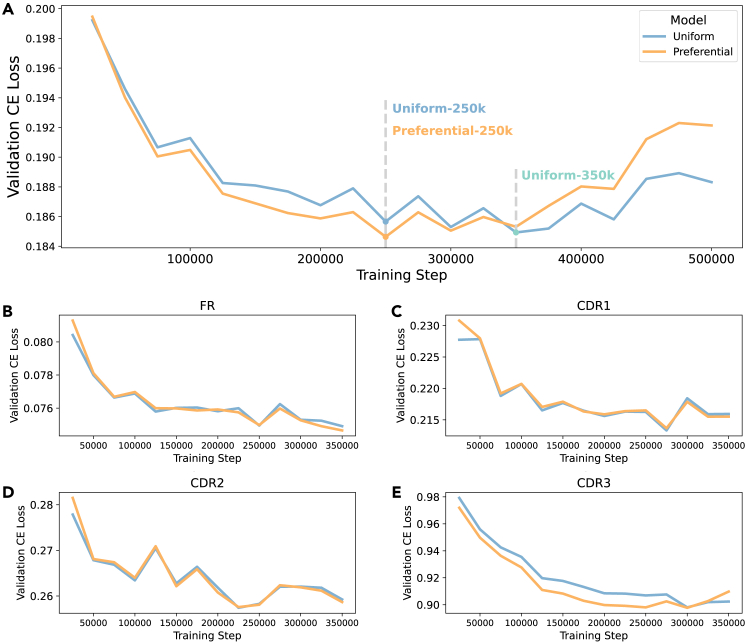
Pre-training validation loss curves (A) Base model validation loss over the course of pre-training. Model checkpoints chosen for downstream analyses are indicated by the dashed lines. (B–E) Overall validation loss was separated into FR (B), CDR1 (C), CDR2 (D), and CDR3 (E) to examine progress of each region. The optimal checkpoints at 250,000 and 350,000 steps were used for downstream analyses, so regional loss plots are limited to the relevant training steps.

The validation loss of the preferential masking model converged with 29% less training time than the uniform model (Figure 2A), suggesting that preferential masking enables learning of the same amount of information with fewer passes through the data. To identify whether improvements in CDR learning were driving this change, validation loss was separated by antibody region: framework (FR), CDR1, CDR2, or CDR3. In all regions, the minimum loss of the preferential model was comparable with or less than that of the uniform model (Figures 2B–2E), with the CDR3 loss additionally converging earlier (Figure 2E). This suggests that preferential masking allows the model to extract more information from the sequence and that reduced masking frequency outside of the CDR3 does not negatively impact representation learning. The optimal checkpoints for the uniform and preferential models are nominally at 350,000 and 250,000 steps, respectively, as the overall validation loss began to increase (Figure 2A), while the training loss continued to decrease (data not shown). This indicates that the optimal uniform model likely falls between 325,000 and 375,000 training steps, or 75,000–125,000 more steps (∼40%) than required for the optimal preferential model.

In the subsequent analyses, we evaluate the uniform and preferential masking models through two comparisons. First, we assess the models at their respective lowest overall validation losses: the uniform model at 350,000 steps (Uniform-350k) and the preferential model at 250,000 steps (Preferential-250k). Second, we compare the models trained with the same computational resources: Preferential-250k versus Uniform-250k.

### Preferential masking improves representation learning of mutated sequences

Antibody sequence diversity is highest in the CDR3; this region contains the junctions between germline V(D)J segments, and non-templated sequence addition and deletion produce variable lengths and increased complexity.^16^^,^^19^ Preferential masking amplifies training signals from the CDR3, so we expect better learning of non-templated patterns when compared to the conventional uniform strategy.

**Figure 3:**
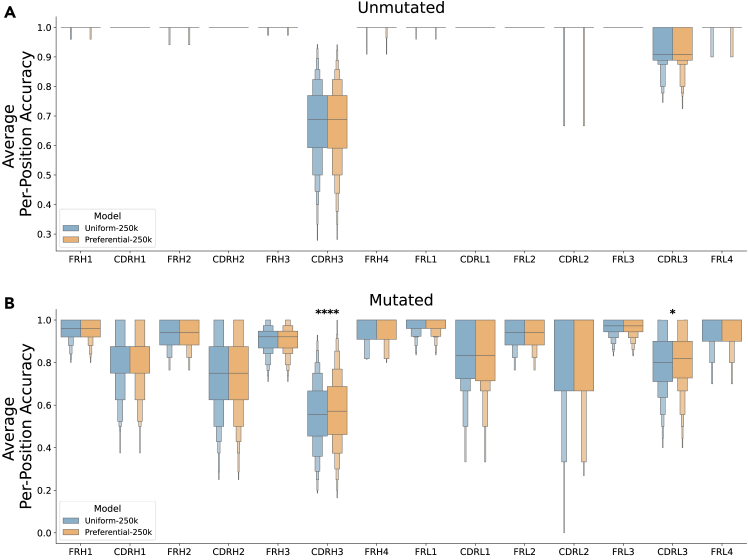
Per-position residue prediction accuracy For 1000 unmutated (A) and mutated (B) test sequences, each residue was iteratively masked and predicted by both models (Uniform-250k and Preferential-250k). Mean prediction accuracy is plotted for each FR and CDR. Statistical significance for each region was calculated using a two-sided paired t test with Bonferroni correction for multiple testing (14 regions). See also Figure S1.

Step-matched model performance was assessed by separately analyzing the per-position prediction accuracy of 1,000 unmutated and mutated sequences from dataset B. Grouping predictions by their corresponding FR or CDR revealed stronger model performance on unmutated sequences (Figure 3A) than mutated sequences (Figure 3B). Similarly, performance in the FRs was stronger than in the CDR3s for both sequence types. These observations are consistent with previous findings that germline-encoded features are more readily learned by LMs than the complex sequence patterns found in primarily non-templated regions.^12^^,^^16^ After Bonferroni correction for multiple testing (14 regions), the Preferential-250k model performed significantly better than the Uniform-250k model at predicting residues in the mutated CDRH3 (*p* = 2.53 × 10^−9^) and CDRL3 (*p* = 3.52 × 10^−3^) (Figure 3B), suggesting that preferential masking is better at recovering predictions of somatic mutations (introduced during affinity maturation). Though residues in the conserved FRs were masked at a lower rate, we observed no significant decrease in performance, indicating that largely germline-encoded regions can be effectively learned with less training signal. The uniform model with 40% more training (Uniform-350k) reaches the Preferential-250k model’s performance in the CDR3 and performs slightly better in some non-CDR3 regions (Figure S1).

### Productive VH/VL pairing is informed by learnable, non-random patterns

Antibody heavy and light chains independently undergo V(D)J recombination before their variable domains, VH and VL, respectively, pair to form a dimer. The interaction between the VH and VL is important for determining antibody stability and binding kinetics,^3^ as the antigen-binding region is composed of CDR loops from both chains.^19^ VH/VL pairing is thought to be largely random, and pairing preferences are not well understood.^3^^,^^26^ Given the need for structural compatibility between the VH and VL, preferential masking of the CDR3s, which are structurally proximal and central to the binding pocket, may lead to an improved understanding of features that determine productive VH/VL pairs.

To assess this, we trained a sequence classification head to identify native vs. shuffled VH/VL pairs. We performed this test on two class-balanced datasets: dataset C (∼65,000 sequence pairs) and dataset D (∼140,000 sequence pairs).

**Figure 4:**
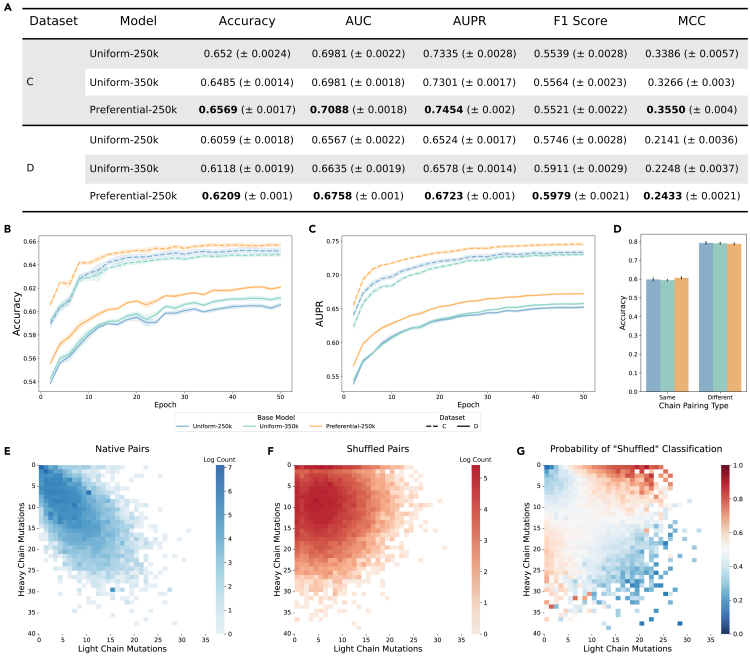
Native vs. shuffled pair classification and chain-specific mutation count analysis (A) Test set metrics for pair classifier models at the end of training. (B and C) Accuracy (B) and AUPR (C) over the course of classifier head training. (D) Accuracy for dataset C separated by chain pairing type. Heavy and light chains are either both unmutated or mutated (same), or one chain is mutated while the other is unmutated (different). All data are represented as mean ± SE for 5 independent training runs using 5-fold CV, and the highest values that are statistically significant for each dataset are bolded in the table. (E and F) Log counts of true native pairs (E) and true shuffled pairs (F) from dataset D for each combination of chain-specific mutation counts. (G) Likelihood of “shuffled pair” classification (softmax of the model output logits) for 60,000 dataset D test sequences by classifiers trained on top of the Preferential-250k base model. More confident predictions are darker, with predicted native pairs in blue and shuffled pairs in red.

All models classified native vs. shuffled pairs with over 60% accuracy, indicating that at least some factors governing VH/VL chain pairing are non-random. The Preferential-250k model outperformed both uniform models across metrics (accuracy, area under to receiver operating characteristic curve [AUC], area under the precision-recall curve [AUPR], and Matthews correlation coefficient [MCC]) (Figure 4A) and maintained superior performance throughout training (Figures 4B and 4C), suggesting that its embeddings were more informative for identification of native chain pairings. Most absolute metrics were higher for the classifier models trained on dataset C, possibly due to inherent similarities to the pre-training data (dataset A), as described in the Methods. In addition, this dataset included both naive (unmutated) and memory (mutated) B cell sequences. Given that AbLM embeddings have been shown to primarily differentiate sequences by mutation,^12^^,^^13^ we anticipated that shuffled pairs containing one unmutated and one mutated chain would be easily distinguishable. To explore this, dataset C predictions were split into two groups: “same” (both chains unmutated or mutated) and “different” (only one chain mutated). Prediction accuracy was notably higher for the different pairs (Figure 4D), suggesting that obvious mutation-based mismatches may have skewed shuffled pair predictions. Dataset D, composed of only memory B cell sequences from donors that were not included in dataset A, was designed to remove these most obvious shuffled pairs, and the increased difficulty of this task is reflected by the lower performance metrics. However, the preferential masking model’s improved performance still holds, suggesting that productive VH/VL pairing is at least somewhat informed by patterns in the CDR3 and that the increased pre-training signal from this region improved learning of these patterns.

### Mutation-driven comparison between heavy and light chains drives native pair classification

Antibody heavy and light chains are paired before undergoing affinity maturation,^2^^,^^3^ in which the VH and VL co-mutate and typically accumulate a similar number of mutations (Figure 4E). By contrast, shuffled pairs adopt a wider mutation distribution between paired chains (Figure 4F). Based on our previous observation that mutation counts contribute to the model’s ability to differentiate native and shuffled pairs, we analyzed the predicted probability toward the “shuffled pair” class for 60,000 test sequences from dataset D (Figure 4G). Heavy- and light-chain pairs with highly dissimilar mutation counts, particularly those with a more heavily mutated light chain, were likely to be classified as “shuffled” with high confidence (shown in red). High confidence “native” classifications (shown in blue) tended to occur when both chains were either unmutated or highly mutated. These observations align with the true mutational distributions of native and shuffled pairs (Figures 4E and 4F). As mutation information is not explicitly provided during training, this separation of classification probabilities by chain-specific mutation level indicates that the base model embeddings contain information about affinity maturation-driven deviation from the germline and, further, that comparative mutation counts between chains are a key driver of native pair classification.

### Pre-training with preferential masking improves binding specificity classification

Previous studies have shown that AbLMs are able to classify sequences by antigen binding specificity.^12^^,^^14^ Though the antibody repertoire is very diverse, antibodies frequently utilize recurring sequence motifs to target a specific epitope, even across individuals.^14^ These motifs are often located in the CDR3 because of its longer loop structure, high variability, and accumulation of mutations during affinity maturation.^16^^,^^19^ Given the critical role of the CDR3 in the antibody-antigen interaction, preferential masking of this region may enhance the model’s ability to classify sequences by antigen binding specificity.

To explore this, we trained a sequence classification head on dataset E (∼25,000 paired sequences) for binary classification of antibodies as SARS-CoV-2 (CoV) specific or not. The negative set (non-CoV specific) was composed of randomly selected memory B cell sequences obtained pre-2020 from several healthy donors to minimize false negative classifications.

**Figure 5:**
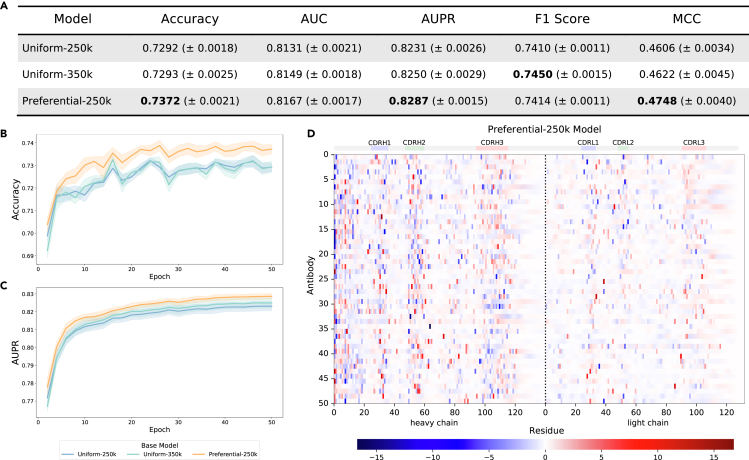
CoV specificity binary classification and AttCAT analysis (A) Test set metrics for CoV classifier models at the end of training. (B and C) Accuracy (B) and AUPR (C) over the course of classifier head training. All data are represented as mean ± SE for 5 independent training runs using 5-fold CV, and the highest values that are statistically significant are bolded in the table. (D) Normalized AttCAT impact scores with respect to the correct label class for 50 systematically chosen test sequences for the CoV specificity classifier trained on the Preferential-250k base model. Top color bars indicate approximate CDR location. Sequences are sorted in ascending order by average prediction probability across the classifiers trained on all 3 base models. High-impact residues are darker, with signal toward the correct label class in blue and away in red. See also Figure S2.

We observed that the Preferential-250k model outperformed both uniform models on most metrics (Figure 5A), highlighting the importance of the CDR3 in determining binding specificity. This improvement was consistent over the course of training (Figures 5B and 5C), suggesting that the output embeddings by the preferential masking base model were more informative for specificity classification than those from the uniform models.

### Models identify residues in the CDRs as important for determining binding specificity

One major limitation of LLMs in both the NLP and protein spaces is interpretability.^27^ As models grow in complexity and are increasingly used to make healthcare decisions, it is important to develop XAI methods to enable better human understanding of model reasoning. We sought to understand the classifier models’ decision-making process for identification of CoV-specific sequences. Since the antigen-binding region is largely composed of the CDR loops, we expect that residues in the CDRs would be identified as most salient for determining binding specificity.

A common method of explaining a transformer’s output is to plot relative activation over the input tokens using raw attention weights from single or multiple layers. While this technique has been applied to existing AbLMs,^12^^,^^15^ it does not consider information flow via other paths^28^ and does not consistently correlate with model performance or other feature importance indicators.^29^ Thus, we used the gradient-based XAI technique Attentive Class Activation Tokens (AttCAT)^29^ to explore which residues were identified by the model as “important” for classifying a sequence as CoV specific.

AttCAT impact scores were computed for 50 systematically chosen test sequences from dataset E for each classifier model with respect to the correct label class (Figures 5D and S2). Negative scores (shown in blue) indicate impact toward the correct label class, while positive scores (shown in red) indicate impact away from the correct label class. High-impact residues, as indicated by score magnitude (darker colors), were concentrated in the CDRs with stronger activation in the heavy chain, suggesting that the models are using residues in these key regions to determine binding specificity. This is consistent with previous XAI analyses on PLMs, which showed that model-identified "important" sequence regions were correlated with known functional regional annotations.^30^ Tokens with strong activation at the beginning of the sequence may represent an “attention sink,” which stores extra attention scores that do not contribute to classification decision-making.^31^

## Discussion

Advancements in NLP have been applied to PLMs and AbLMs with little adaptation of the methods to accommodate the unique features of biological data. While this approach has enhanced our understanding of general structure and function, these models fall short in capturing the highly variable CDR3, which plays a crucial role in antigen binding. Here, we sought to improve CDR3 understanding by implementing an antibody-specific modification to the pre-training strategy.

We present preferential masking as a compute-efficient approach for antibody representation learning, achieving similar predictive performance to uniform masking while reducing training steps by 29% (Figures 2 and 3). By increasing the frequency of masking and prediction in high-diversity regions, we enhance the diversity of the training data seen by the model, leading to greater efficiency. Furthermore, our use of dynamic masking^20^ effectively presents a unique sequence context for each input during every epoch, demonstrating that preferential masking also boosts data efficiency, which is particularly important given the limited scale of training data available for AbLMs.^12^^,^^14^^,^^16^ Preferential masking enhances downstream classification of antibody sequences by native chain pairing (Figure 4) and binding specificity (Figure 5), highlighting the role of the CDR3 in antibody development and function. While these specific binary classification tasks may have limited practical use, our findings suggest that preferential masking improves the learning of immunologically relevant patterns, potentially also enhancing performance on other downstream tasks, such as structure prediction.

Analysis of native vs. shuffled pair classification indicates that antibody VH/VL pairing is not random, contrasting previous studies^26^ and supporting emerging evidence of potentially learnable patterns that influence pairing decisions.^32^ This observation is particularly notable for dataset D, which contains only memory B cell sequences from donors distinct from those used in pre-training, and suggests that non-random pairing is a true biological phenomenon rather than a result of confounding factors. Given that antibody chain pairing is inherently promiscuous, presumably with the evolutionary objective of increasing repertoire diversity,^33^ it may not be possible for an AbLM to reliably predict one-to-one chain pairings. However, insights from these models (Figure 4G) can enable elimination of highly unlikely pairings, which is particularly beneficial for tasks such as directed evolution, where rather than identifying a single “best” sequence, it is more important to remove clearly unnatural pairs that likely express poorly and may have undesirable functional properties like poly-reactivity.

AttCAT analysis reveals that our CoV specificity classifier models are primarily informed by residues in the CDRs, especially on the heavy chain (Figure 5D). The antigen-binding pocket consists of CDR loops from both heavy and light chains, with the CDRH3 being the most diverse and influential in driving specificity.^19^ This indicates that our models have correctly identified the crucial regions for determining binding specificity, demonstrating learning of meaningful immunological patterns and validating our use of LM techniques for antibody sequence modeling.

Entropy or importance-based weighting has been successfully applied to NLP tasks, leading to improved training efficiency and downstream performance.^22^ The CDR3 represents areas of high entropy and was selected for preferential masking due to its functional relevance and significantly higher diversity. Though our approach is limited by the need for data to be annotated with non-templated residue locations, many antibody sequencing pipelines, such as Cell Ranger^34^ and abstar,^35^ already include these annotations. Integrating preferential masking into existing AbLM training is also relatively straightforward and requires minimal additional computation, as masking probabilities can be derived with only a single pass through the dataset. However, a more sophisticated weighted masking strategy could further enhance efficiency and reveal additional significant areas for improving antibody representation learning. Although non-templated residues are primarily found in the CDR3, they are not exclusive to this region; other existing sequence annotations, such as mutations resulting from affinity maturation, could be effective targets of preferential masking. Further, a dynamic, data-driven strategy could make this process entirely self-supervised. For example, focal loss, which adjusts token-specific impact on the loss at each pre-training step, has been explored for AbLMs and shown to increase out-of-distribution predictions.^16^ A similar approach could be adapted for weighting the masking probability.

Our classifier model analyses indicate that there are learnable, sequence-inherent patterns that can effectively differentiate groups of antibodies. A better understanding of these patterns could deepen our knowledge of the fundamental immunological properties governing antibody development and function. However, deciphering the decision-making processes of LMs remains challenging. This study is among the first^14^ to apply post hoc XAI techniques to AbLMs, illustrating how LM-learned patterns can become interpretable. While AttCAT offers a high-level overview of information flows and learned parameters, it lacks the granularity needed to extract detailed patterns. Additionally, because AttCAT is designed for classification models, its insights are limited to task-specific contexts and do not fully capture the knowledge acquired by the underlying pre-trained models. Further work on XAI tailored to biological data is needed to enable discovery of novel patterns from complex LMs.

There is still significant potential for improving AbLMs. These models are currently trained on relatively small datasets compared to state-of-the-art LMs for NLP. Expanding the training data via additional sequencing or mining of databases like the Observed Antibody Space^36^ could enhance the diversity of learned patterns and improve generalizability. In addition to BERT-based AbLMs, autoregressive AbLMs^37^^,^^38^ have been explored for antibody design tasks. These architectures may also benefit from immunologically informed pre-training, similar to what is presented here. Furthermore, incorporating additional modalities may enhance model performance. Models like ESM3, for example, which combine sequence data with structural and functional information, have demonstrated improvements in generative capabilities.^39^ Given that AbLMs outperform general PLMs in antibody-specific tasks,^11^^,^^12^^,^^15^ a multimodal AbLM could greatly expand our understanding of antibody-specific features and broaden the potential applications of these models.

In summary, we present three key findings that will help inform future development and analysis of AbLMs. First, preferential masking of the primarily non-templated CDR3 efficiently enhances antibody representation learning, demonstrating that using biological knowledge to inform LM training is a promising strategy for improving protein modeling. Second, VH/VL pairing is governed by learnable, non-random, sequence-inherent patterns, which could have implications for antibody engineering. Finally, the application of XAI highlights the fact that AbLMs are capable of learning meaningful biological patterns. XAI enables interpretation of these complex patterns, offering potential insights into the fundamental immunological properties that underlie antibody development and function.

## Methods

### Preferential masking implementation

The dynamic masking step of MLM is implemented in the HuggingFace^40^ data collator, where, for each tokenized input sequence, a probability matrix is generated with the masking probability for each token index. The conventional probability matrix is a sequence-length vector of 0.15, which uniformly masks the entire sequence at 15%. For preferential masking, we first construct a CDR mask for each sequence to reflect the region (FR or CDR1–CDR3) in which the residue resides. Then, the masking probability $P(x_{m}$) for each residue x was calculated using the following equation:

$$P\left(x_{m}\right)=\left\{ \begin{array}{rr} 0.25, & x\in CDR3\\ \frac{0.15-0.25{\hat{p}}_{CDR3}}{1-{\hat{p}}_{CDR3}}, & x\notin CDR3 \end{array}\right.\text{,}$$

where ${\hat{p}}_{CDR3}$ refers to the proportion of residues in the sequence that reside in the CDR3. The CDR3 masking rate of 25% was chosen based on initial small-scale pilot tests (data not shown), which revealed that masking CDR3 residues at approximately twice the frequency of non-CDR3 residues yielded the greatest improvement in overall validation loss. Further increases in the masking rate likely resulted in too little CDR3 context to inform predictions and, thus, decreased performance. To verify this, a full-size AbLM was also pre-trained using a CDR3 masking rate of 35%. It was similarly observed to overfit faster than our other models and showed no significant improvements in downstream performance over the model pre-trained using 25% (Figure S3).

For comparison with the conventional MLM strategy, an average masking probability of 15% across the entire sequence length was maintained. This is to ensure that comparisons are due to the modified masking strategy and not confounding factors such as increased overall masking. Construction of the CDR masks and subsequent calculation of preferential masking probabilities was done in a single initial pass over the entire dataset to eliminate redundant computation during model pre-training.

### Datasets

Table 1 gives a brief overview of the datasets used in this paper. Antibody sequences in these analyses refer to only the variable domains (VH/VL). Details on dataset construction can be found below.

**Table 1:** Dataset overview

Dataset	Sources	Description of Use	Figures
A	Jaffe et al.^32^ Hurtado et al.^41^	Base model pre-training (training and validation sets)	Figures 2 and S3
B	Jaffe et al.^32^ Hurtado et al.^41^	Base model testing (per-residue inference)	Figures 3, S1, and S3
C	Jaffe et al.^32^ Hurtado et al.^41^	Pair classification (same donors as pre-training, made by shuffling dataset B)	Figure 4
D	Phad et al.^42^ Data generated as part of this project	Pair classification (memory sequences from distinct donors from pre-training)	Figure 4
E	Raybould et al.^43^ Phad et al.^42^	CoV specificity classification and AttCAT analysis	Figures 5, S2, and S3

#### Base model pre-training: Datasets A and B

For model pre-training, we used the largest publicly available dataset of natively paired antibody sequences, approximately 1.6 million pairs, from Jaffe et al.^32^ These sequences were isolated from circulating B cells from healthy adult human donors (*n* = 2), and were not enriched for any particular antigen binding. We supplemented this original dataset with ∼400,000 paired B cell sequences from the control dataset of Hurtado et al.,^41^ which were also isolated from circulating B cells from healthy adult human donors (*n* = 15). Raw sequences were annotated with abstar^35^ to extract the amino acid sequence of each V(D)J region. Abstar uses ImMunoGeneTics (IMGT) numbering to annotate CDRs.^35^ Label-encoded masks of the CDRs were constructed using the abutils package^35^ via semi-global alignment of V(D)J annotations to the full amino acid sequence. The data were filtered to remove duplicates and CDR mask alignment errors, such as incorrectly placing multiple CDRs adjacently in the sequence. The data were then clustered at 96% identity, resulting in 1,622,679 sequence pairs. The data were randomly split into training/validation/test sets at a ratio of 92:4:4, respectively, meaning that all donors are represented in all splits. The training and validation splits, which we refer to as dataset A, were used for pre-training of the base models. The test split, which we refer to as dataset B, was used for per-position inference.

#### Native pair classification: Datasets C and D

For the native pair classification task, healthy donor antibody sequences (not enriched for any particular antigen) were clustered at 95% identity, and non-native pairs were generated by sampling 50% of the sequences from each donor and randomly shuffling the heavy and light chains.^12^ Balanced classes for native vs. shuffled pairs allows for the splitting of training and test sets by donor. Due to light chain redundancy, a very small percentage (<0.1%) of the shuffled pairs were native; these were filtered out. The data were randomly split using a 5-fold cross-validation (CV) with stratification.

Two native vs. shuffled pair datasets were created for this task. Dataset C contains naive and memory B cell sequences from the same donors as the pre-training data and was created by shuffling dataset B, resulting in 64,874 sequence pairs (32,437 of each class). Dataset D contains only memory B cell sequences from different donors than dataset A and was created by shuffling paired memory B cell sequences obtained from Phad et al.^42^ (*n* = 2) and our own sequencing of peripheral blood mononuclear cells (PBMCs) from healthy donors (*n* = 3), resulting in 146,668 sequence pairs (73,334 of each class).

#### CoV-specificity classification: Dataset E

For the specificity classification task, CoV antibody sequences were obtained from CoV-Ab-Dab (downloaded June 13, 2023).^43^ The negative set consisted of paired memory B cell sequences from Phad et al.^42^ that were obtained pre-2020 to minimize the occurrence of false negative classifications. Amino acid sequences were clustered at 95% identity, resulting in 24,970 total sequence pairs (12,485 of each class), which we refer to as dataset E. The data were randomly split using a 5-fold CV with stratification.

### Base model pre-training

We separately trained 2 models on dataset A using an MLM objective: one using conventional uniformly random masking and one using preferential masking. In both masking strategies, tokens were independently selected for prediction with an average probability of 15% across the entire input sequence length. From these, 80% are replaced with a mask token <mask>, 10% are replaced with a random token from the vocabulary, and the remaining 10% is left unchanged.^18^ Masking was performed dynamically^20^ to avoid encountering the same mask across epochs. For the preferential masking model, construction of the CDR masks and calculation of masking probabilities was done in a single initial pass over the entire dataset, stored, and reused in each epoch to eliminate redundant computation during model pre-training. Both models use an encoder-only ESM-2 architecture^10^ with 32 layers, 20 attention heads per layer, a hidden size of 960, and an intermediate size of 3,840, resulting in both models having ∼350 million parameters.

The vocabulary contained 26 tokens: 1 for each of the proteinogenic amino acids, “X” for unnatural amino acids, and 5 special tokens: <pad>, <mask>, <unk>, <cls>, and <eos>. Inputs were heavy- and light-chain sequences concatenated by two <cls> tokens (Figure 1) and padded to length 320 to accommodate the length of the longest sequence pair without truncation.

Both models were implemented using a slightly modified HuggingFace transformer library^40^ and trained using DeepSpeed.^44^ Both models were trained using a total batch size of 256 for a total of 500,000 steps (∼85 epochs) on 8 NVIDIA A100 graphics processing units, equating to ∼52 h per model. The learning rate increased linearly to a peak of 1e−4 over the first 30,000 steps and decayed linearly thereafter.

The pre-training task of the preferential masking model was effectively harder than that of the uniform model, as increased CDR3 masking results in the prediction of more “difficult” residues. Therefore, to compare training progression and monitor overfitting, validation loss for both models was calculated using cross-entropy loss under the uniform masking strategy. For practical reasons, model checkpoints were only saved every 50,000 steps. Overfitting was observed in both models (Figure 2A), so the available optimal checkpoints at 250,000 steps (∼43 epochs) and 350,000 steps (∼60 epochs) were chosen for downstream analyses.

### Classifier model training

A sequence classification head (single feedforward layer) was trained for binary classification of native vs. shuffled chain pairing (datasets C and D) or CoV-specific vs. healthy donor antibodies (dataset E). Since we desire to evaluate the pre-training strategy via downstream applications of its output embeddings, the base model weights were frozen so as to not alter those embeddings during classifier head training. Sequences were tokenized using the same tokenizer as for pre-training, and no truncation was necessary, as all sequences were shorter than the model’s maximum input length. Classifier models were trained for 50 epochs with a total batch size of 256, a peak learning rate of 1e−5, and a linear warm-up ratio of 0.1. For each classification task, 5 different shuffled, stratified splits of the data were generated using 5-fold CV, and a classifier head was trained independently on each of these splits for all base models. Training with a 5-fold CV was chosen to show variation based on the training/testing data. We ensured that all inference was performed by models that did not include the corresponding sequence in its training set.

Binary classifiers were evaluated using the following metrics: accuracy, AUC, AUPR, F1 score, and MCC. Test set accuracy and AUPR plots are averaged across 5 independent training runs with standard error. Metrics were calculated using scikit-learn and are based on Weights & Biases (wandb.ai) logging data. Analyses involving mutation counts were conducted using abstar^35^ annotations of V gene amino acid mutations.

### Specificity classifier model interpretation

AttCAT is a gradient-based attribution XAI method developed by Qiang et al.^29^ for visualizing which input tokens most inform a classification decision. AttCAT uses gradient information from all model layers and heads to quantify token impact and is adapted for the transformer architecture by integrating self-attention weights to capture global contextual information. The output is a set of “impact scores” that quantifies the informativeness of each input token for a specific target class. We chose this XAI method over other feature attribution-based methods or attention alone because AttCAT incorporates features, gradients, and self-attention into its score, giving a more comprehensive evaluation of all information flows through the model. Scores were normalized using standard scaling to facilitate comparison between models. Code was adapted from Song et al.^28^ to work with the ESM model architecture.

### Antibody sequencing

De-identified PBMCs from healthy donors (*n* = 3) were first enriched for memory B cells using the EasySep Human Memory B Cell Isolation Kit (STEMCELL Technologies, catalog number 17864), sequenced using the 10x Genomics pipeline, and analyzed using Cell Ranger and custom Python scripts as described by Hurtado et al.^45^

## Resource availability

### Lead contact

Requests for further information and resources should be directed to and will be fulfilled by the lead contact, Bryan Briney (briney@scripps.edu).

### Materials availability

This study did not generate new unique reagents.

### Data and code availability

All code necessary to reproduce the results and figures in this paper is available through GitHub (github.com/brineylab/preferential-masking-paper). All data,^46^ including model weights and sequence datasets used for model training, validation, and testing, are available through Zenodo: https://doi.org/10.5281/zenodo.13973759.

## Acknowledgments

The authors would like to thank Jonathan Hurtado and Simone Spandau for help with generating sequencing data (dataset D) and Kenney Ng for helpful feedback on early manuscript drafts. This work was funded by the 10.13039/100000002National Institutes of Health (P01-AI177683, U19-AI135995, R01-AI171438, P30-AI036214, and UM1-AI144462) and the Pendleton Foundation. K.N. is a recipient of the Endowed Fellowship in the Skaggs Graduate School of Chemical and Biological Sciences.

## Author contributions

Conceptualization, K.N. and B.B.; model training and evaluation, K.N.; manuscript preparation and revisions, K.N. and B.B.

## Declaration of interests

B.B. is a shareholder of Infinimmune and a member of its scientific advisory board.

## Supplemental information

Document S1. Figures S1–S3

Document S2. Article plus supplemental information
