# Supplementary material for: Focused learning by antibody language models using preferential masking of non-templated regions
Source: Patterns (N Y). 2025 Apr 25;6(6):101239. doi: 10.1016/j.patter.2025.101239 (PMC12191730; doi:10.1016/j.patter.2025.101239)
Supplement: Document S1. Figures S1–S3 [file mmc1.pdf]

Patterns, Volume *6*

Supplemental information

Focused learning by antibody language models using preferential masking of non-templated regions

Karenna Ng and Bryan Briney

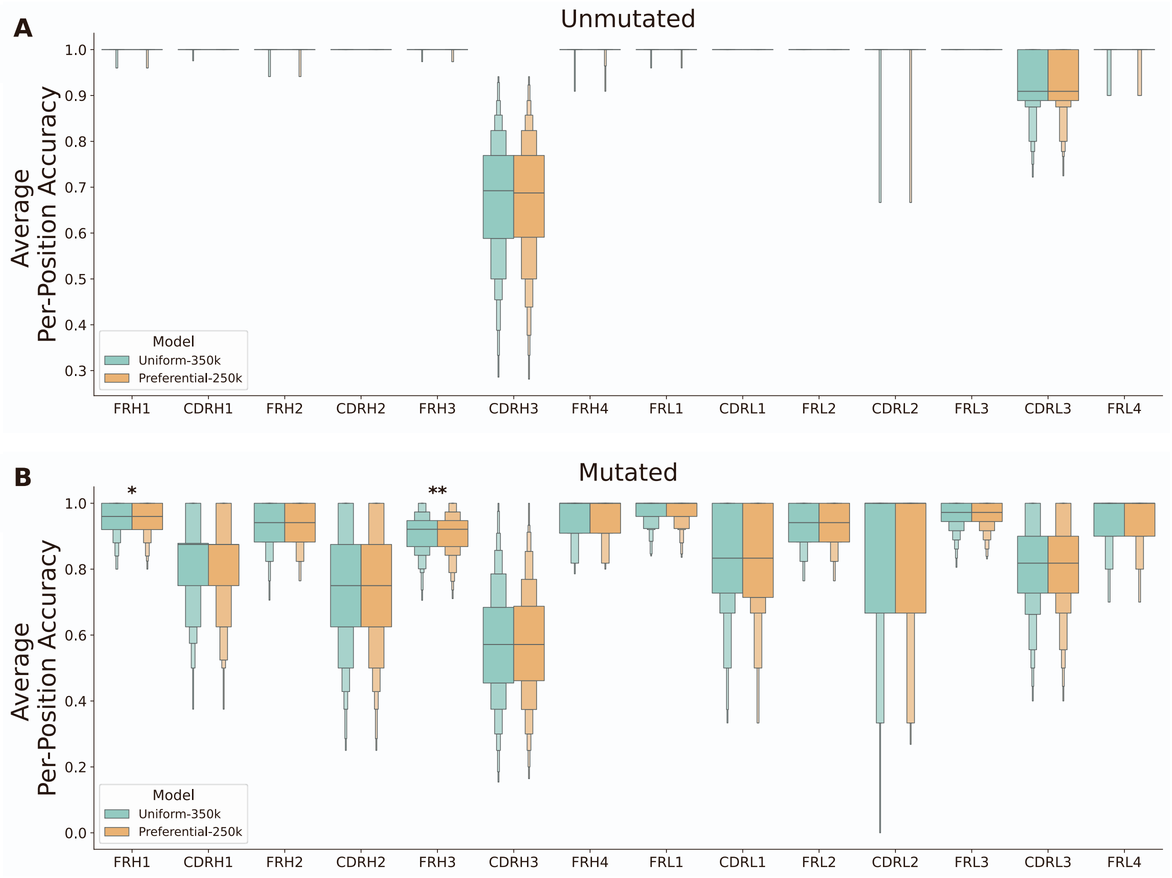

Figure S1. Per-position residue prediction accuracy. For 1000 unmutated (A) and mutated (B) test sequences, each residue was iteratively masked and predicted by both models (Uniform-350k and Preferential-250k). Mean prediction accuracy is plotted for each FR and CDR. Statistical significance for each region was calculated using a two-sided paired t-test with Bonferroni correction for multiple testing (14 regions). Related to Figure 3.

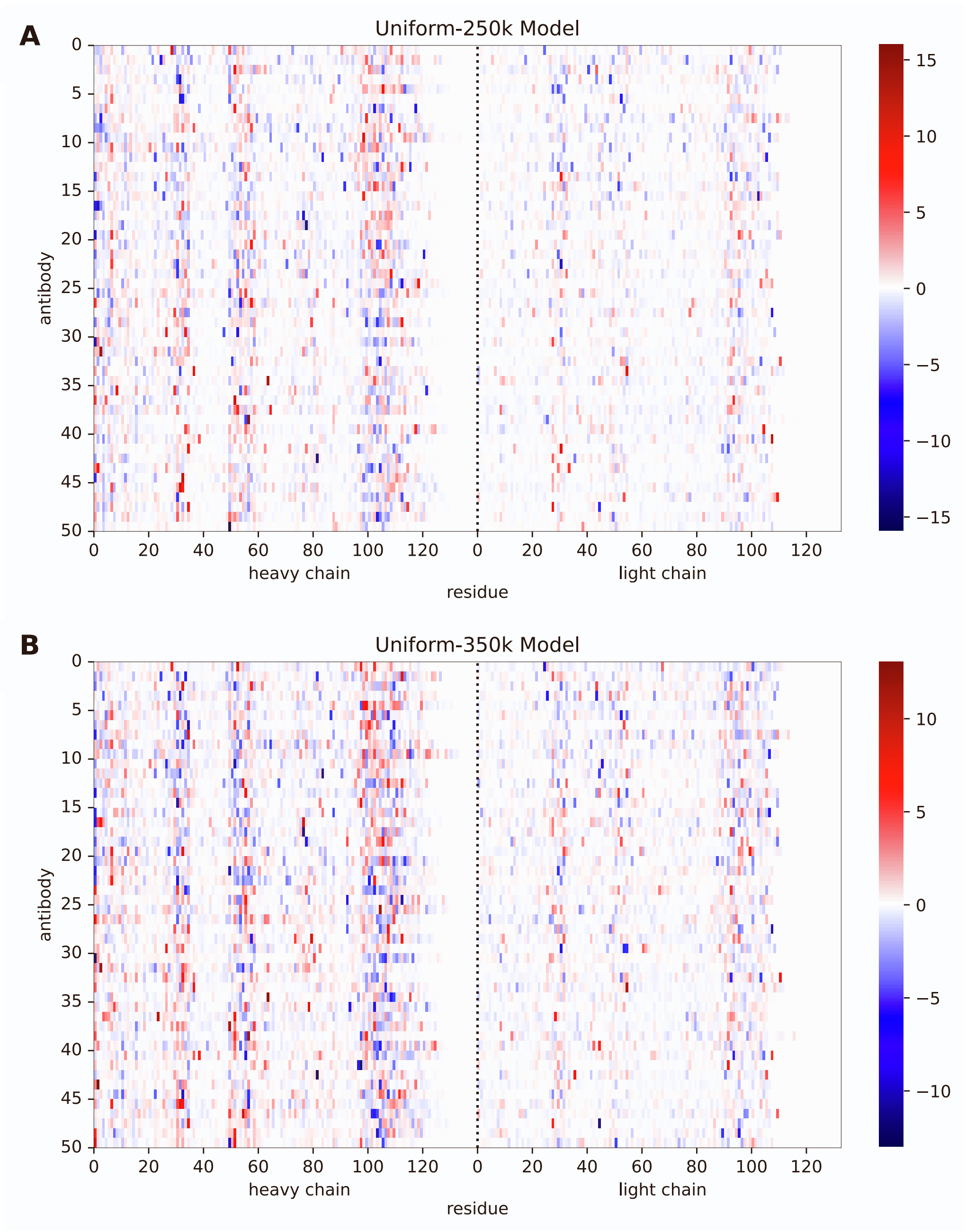

Figure S2. AttCAT analysis on the Uniform-250k and Uniform-350k CoV specificity classifier models. Normalized AttCAT impact scores with respect to the correct label class for the same 50 systematically chosen test sequences for the CoV specificity classifiers trained on the Uniform-250k (A) and Uniform-350k (B) base models. Sequences are sorted in ascending order by average prediction probability across the classifiers trained on all 3 base models. Related to Figure 5.

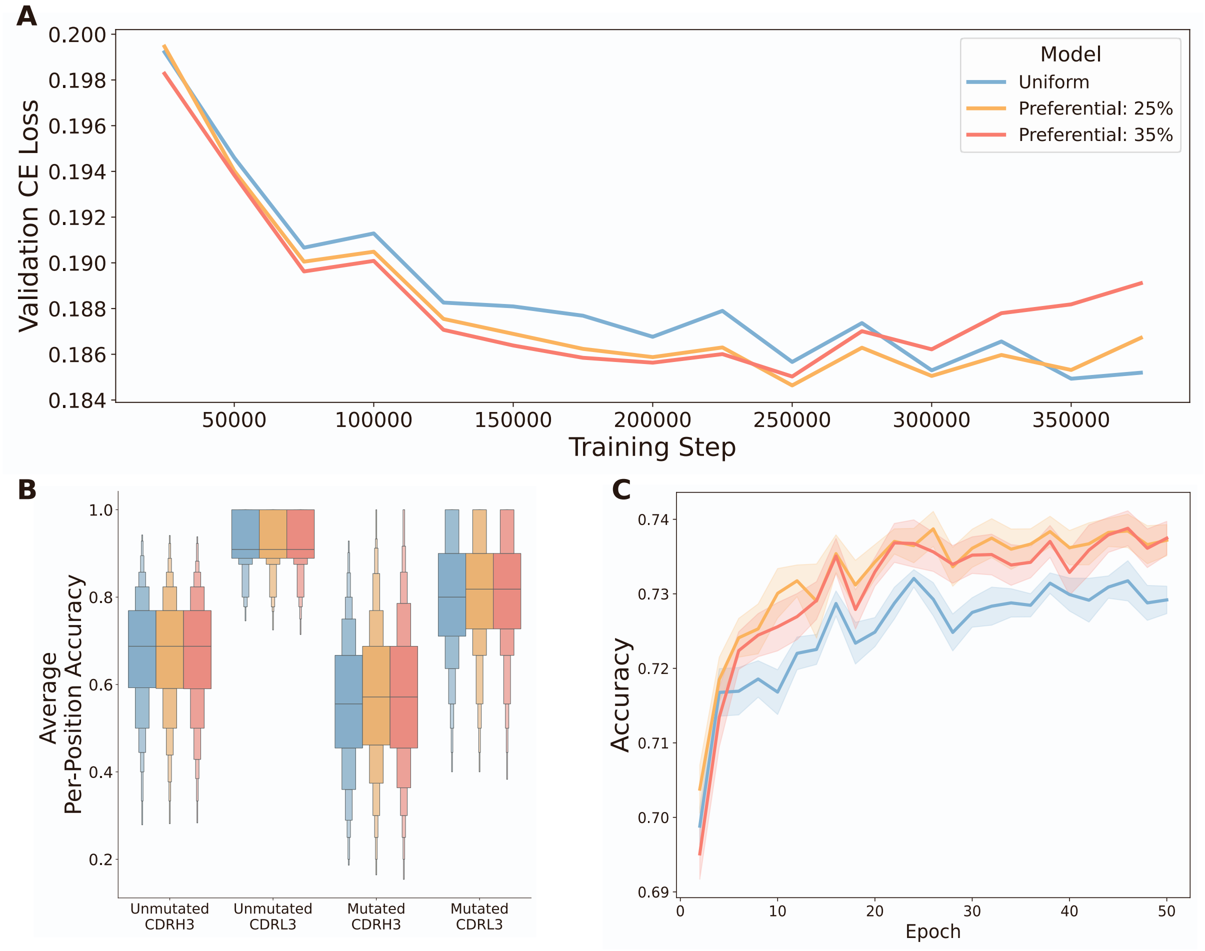

Figure S3. Pre-training with a CDR3 masking rate of 35%. (A) Base model validation loss over the course of pre-training for various CDR3 masking rates (15%, 25%, and 35%). Subsequent panels use the checkpoints at 250,000 steps. (B) Mean per-position prediction accuracy on 1000 test sequences for the unmutated and mutated CDR3s. (C) Accuracy over the course of CoV classifier head training. Mean ± SE is shown for 5 independent training runs using 5-fold CV.
